# Correlations between Poor Micronutrition in Family Members and Potential Risk Factors for Poor Diet in Children and Adolescents Using Korean National Health and Nutrition Examination Survey Data

**DOI:** 10.3390/nu7085286

**Published:** 2015-08-04

**Authors:** Hye Ah Lee, Hyesook Park

**Affiliations:** Department of Preventive Medicine, School of Medicine, Ewha Womans University, 1071, Anyangcheon-ro, Yangcheon-ku, Seoul 158-710, Korea

**Keywords:** children, diet, family study, correlation, micronutrients

## Abstract

Based on data from the 2010–2011 Korean National Health and Nutrition Examination Survey, we investigated correlations between micronutrients in the diet of family members and the possible risk factors for children and adolescents consuming an inadequate diet. We examined two-generation households with children aged 2–18 years. The quality of the family diet with regard to the following nine nutrients (protein, calcium, phosphorous, iron, vitamin A, vitamin B1, vitamin B2, niacin, and vitamin C) was assessed based on the Index of Nutritional Quality. Correlations between quality of diet and selected variables were analyzed using the Statistical Analysis for Genetic Epidemiology software, and those between diet quality and potential risk factors for poor diet in offspring were analyzed using multinomial logistic regression. Overall, calcium was the most commonly under-consumed micronutrient. More than half of sons and daughters showed insufficient vitamin A, vitamin C, and iron intake, and both mothers and fathers showed insufficiency with respect to vitamin A, vitamin B2, and vitamin C. The correlation between a poor diet in parents and that in offspring was 0.17 (*p* < 0.0001), and this correlation coefficient was higher between mothers and offspring than between fathers and offspring. Additionally, eating breakfast provided a significant protective effect against the risk of poor nutrition in offspring, even after adjusting for covariates. Our results add to evidence indicating that children should be encouraged to eat breakfast to improve the quality of their diet.

## 1. Introduction

Both the quality and quantity of nutritional intake play important roles in health, especially among children and adolescents, who are experiencing a period of growth that has important implications for their ability to achieve their full potential. Unfortunately, most research has focused primarily on total energy intake or consumption of various food groups, likely because obesity is a major public health problem. Interestingly, the total energy intake of Koreans gradually decreased between the late 1960s and 1995, whereas the body size of Korean children and adolescents has increased [1]. Thus, the increase in morbidity and mortality related to obesity and chronic diseases may be partially caused by changes in the composition or quality of nutrients consumed. For example, several recent epidemiological studies showed that insufficient micronutrient (e.g., vitamin D, calcium) intake may be associated with various chronic diseases [2,3] and that dietary fat intake is associated with coronary heart disease [4].

The quality and quantity of the nutritional content of food consumed is influenced by demographic, socioeconomic, environmental, and behavioral factors. Additionally, individuals in the same household share an environment and often eat together, and this is especially true for children, who are affected by their parents’ health-related behaviors [5]. Most, though not all, extant research has supported correlations among the consumption patterns of family members [6]. The majority of family-based studies have focused on macronutrient intake, such as total energy and percentage of macronutrient intake [6]. However, scant research about the similarities and differences in the micronutrient consumption of family members has been conducted. The quality of individuals’ nutritional intake is often measured by comparison with guidelines or national dietary reference data [7,8]. One study of national data from the US reported similarities among the nutritional consumption patterns of family members, but this study also focused on macronutrients [9].

Thus, we investigated the diet of Korean children and adolescents, examining the extent to which their consumption of micronutrients resembled that of family members using data from the Korean National Health and Nutrition Examination Survey (KNHANES). We also explored the possible risk factors for poor diet in children and adolescents, including socioeconomic status, parental smoking, and personal eating behaviors.

## 2. Methods

### 2.1. Study Subjects

Since the KNHANES began in 1998, survey has been performed in 2001, 2005, 2007–2009 and 2010–2012. It is an ongoing annual household-based survey relying on a multi-stage sampling method. The survey is conducted with everyone who is at least one year of age within a given household. Sampling plan before the survey was conducted every wave of the survey. The survey system has been revised from short duration to cover every weeks of the year since 2007 survey. The details of the KNHANES have been described elsewhere [10]. In the present study, data for information on nutritional intake was available from 2010–2011 KNHANES.

To assess correlations among the diets of family members, we limited the sample to two-generation households with offspring aged 2–18 years and classified the age of offspring into three groups: preschool children (2–5 years), children (6–12 years), and adolescents (13–18 years). We excluded individuals who ate less than 500 kcal or more than 5000 kcal in total energy per day, and children for whom information on the nutrition intake of both of parents was missing were also excluded. The study sample included 1283 households (1102 fathers, 1239 mothers, 1020 sons, and 949 daughters) with offspring aged 2–18 years; 25.4% (*n =* 501) were preschool children, 39.5% (*n =* 777) were children, and 35.1% (*n* = 691) were adolescents.

### 2.2. Dietary Data

The KNHANES collected two types of nutrition-related data. The first concerned the foods that participants recalled consuming during a 24-h period, and the second concerned the frequency with which certain foods were consumed (via food frequency questionnaire). This study used the 24-h dietary recall data regarding total food intake during the weekday before the survey, which were collected in interviews conducted by trained dietary interviewers. The quality of respondents’ diets was assessed using the index of nutritional quality (INQ), which is based on the nutritional density per 1000 kcal. The INQ is calculated by dividing the nutritional intake per 1000 kcal of total energy intake by the recommended intake (RI) of each nutrient per 1000 kcal. Age- and sex-specific RI values were obtained from the 2010 Korean Dietary Reference Intake (KDRI) [11]. Each INQ value ≥1.0 indicates adequate nutritional intake, which we defined as indicating sufficient nutritional intake; values <1.0 indicated inadequate nutritional status. The INQ can reveal that intake of a specific nutrient is insufficient even if individuals have reached their total energy requirement. The number of nutrients for which intake was insufficient was used as a measure of diet quality; therefore, it score ranged from 0 to 9. Higher values reflect insufficient intake of a greater number of nutrients. Based on a review of previous related studies [12,13], we selected nine nutrients for examination: protein, calcium, phosphorous, iron, vitamin A, vitamin B1, vitamin B2, niacin, and vitamin C.

### 2.3. Socioeconomic Characteristics, Parental Smoking, and Personal Eating Behaviors

Socioeconomic status was based on parental education and household income. Parental education was classified into two groups: no more than a high school degree (≤12 years of schooling) and college degree or higher (13 years or more of schooling). Household income was defined by dividing the total monthly household income by the square root of the number of individuals in the household; these data were then categorized into quartiles. Finally, a current parental smoking habit was considered to reflect unhealthy behavior and, consistent with a previous study [5], was defined as either smoking at least 100 cigarettes during their lifetime or currently smoking every day or some days.

Eating breakfast, eating with family members, frequency of eating with family members per day, eating breakfast with family members, eating dinner with family members, and frequency of eating out were used as indicators of eating behavior. Regularly eating breakfast was defined as having eaten breakfast on both days before the survey. Eating with family members was defined as at giving least one affirmative answer to questions about eating breakfast, lunch, and dinner with family members. Frequency of eating with family members was categorized into three groups: twice per day, once per day, and never. Based on the distribution of responses, the frequency of eating out was classified into two groups: at least once per day and none.

### 2.4. Statistical Analysis

Statistical analyses were performed with SAS version 9.3 (SAS Institutes, Cary, NC, USA). We used statistical modules (e.g., PROC SURVEY~) that were appropriate for the complex sampling design of the survey. Continuous variables are expressed as weighted means, and categorical variables are expressed as weighted percentages. All statistical calculations followed the data analysis guidelines provided by KNHANES.

Correlations between insufficient nutrient intake and poor diet quality were examined using the FCOR program in the Statistical Analysis for Genetic Epidemiology software package [14]. With regard to the interpretation of the correlation coefficient, we followed a study conducted by Wang *et al.* [6]. By the range of correlation coefficient, 0.10 ≤ | r | < 0.30 are regarded as weak, 0.30 ≤ | r | < 0.50 as moderate, and | r | ≥ 0.50 as strong association. We also conducted a sensitivity analysis, which excluded the data from the relatively few subjects with diseases related to dietary control. Specifically, 70 fathers and 48 mothers reported dietary control due to hyperlipidemia, hypertension, diabetes, thyroid disease, gastric disease, and other reasons; seven children had been dieting due to dodecadactylitis pyelitis or atopy.

Poor diet quality was categorized according to the number of insufficient nutrients as low (0–3), middle (4–6), or high (7–9). The quality of the diet of offspring by the socioeconomic status of the household parental smoking and personal eating behaviors were examined using univariate analyses. Adjusted odds ratios (AORs) with 95% confidence intervals (95% CIs) for unhealthy eating behaviors among offspring were calculated using multinomial logistic regression after controlling for the household environment to develop possible strategies for improving the dietary intake of this population. We considered maternal education as a household characteristic because paternal education was significantly associated with maternal education, and mothers usually prepare family meals. Thus, maternal educational level and household income, as reflective of the household environment, as well as sex and age were treated as covariates. All *p*-values were two tailed, and *p*-values <0.05 were considered to indicate statistical significance.

## 3. Results

**Figure 1:**
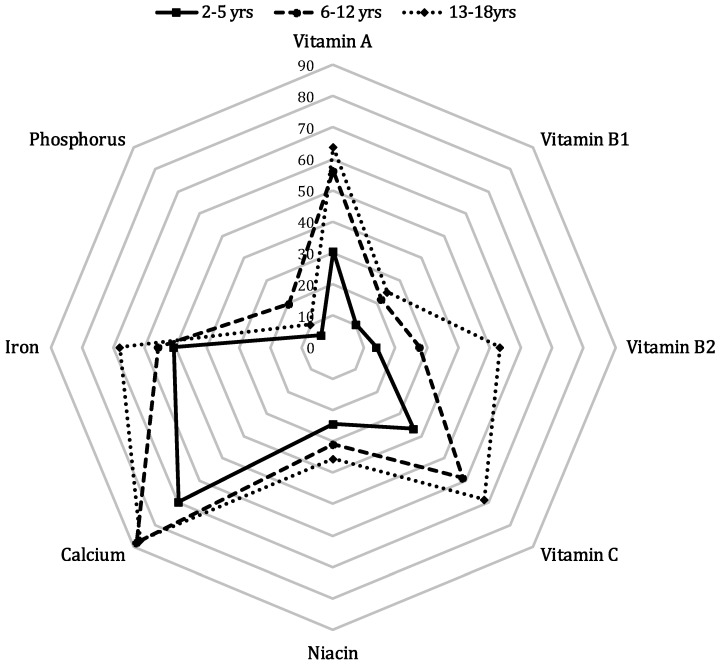
Weighted percentages of Korean children with insufficient nutrient intake by age^. a^ *p* < 0.01 for age differences, ^b^ *p* < 0.0001 for age differences. Intake of each nutrient per 1000 kcal of total energy by age and sex and recommended intake of each nutrient per 1000 kcal. The weighted percentages of those with insufficient intake of each nutrient were calculated in consideration of the sampling method used by the Korean National Health and Nutrition Examination Survey. The weighted percentages of Korean children with insufficient nutrient intake were calculated by age group (squares: 2–5 years; circles: 6–12 years; diamonds: 13–18 years), where indicates all children.

Table 1 presents a summary of the average dietary intake of the sample and the proportion of individuals whose intake of each nutrient and overall diet quality were inadequate. Of the nine nutrients studied, calcium was the most commonly under-consumed nutrient, with insufficient consumption in 74.5% of mothers and 86.7% of daughters. At least half of both sons and daughters consumed inadequate quantities of vitamin A, vitamin C, and iron, and approximately 50% of mothers and fathers consumed insufficient quantities of vitamin A, vitamin B2, and vitamin C. Less than 1% of parents consumed insufficient quantities of phosphorus, but these figures were 10.6% and 14.5% in sons and daughters, respectively. On average, fathers consumed less than the recommended quantity of 2.9 nutrients; these figures were 3.6 among mothers, 3.4 among sons, and 3.9 among daughters. Figure 1 presents the percentages of children with insufficient nutritional intake by age. Overall, the micronutrient intake status was worst in adolescents and best in preschool children. When the data were stratified by sex, we found significant differences for most micronutrients by age; however, vitamin B1, niacine, and iron intake by sons did not differ significantly by age. Insufficient intake of vitamin B1, calcium, niacin, and iron was more common in daughters than in sons, except among preschool children (Figure 1, Tables S2 and S3).

Table 2 presents correlations among the nutritional statuses of family members. The data from parents and offspring regarding insufficient intake were significantly correlated for all nutrients except protein. The correlation coefficient was highest for vitamin C among the total sample, between fathers and sons, and between mothers and daughters. We also found a significant, albeit weak, correlation (*r* = 0.17) between the overall quality of the diet consumed by parents and that consumed by their offspring. When data were analyzed according to the sex of parents and of offspring, the correlation between mothers and offspring was higher than that between fathers and offspring. In terms of the age of offspring, the correlation between the diet of children aged 6–12 years and that of their parents was strongest, and that between the diet of adolescents and that of their parents was weakest. Poor dietary intake among preschool children was significantly correlated with poor dietary intake by their mothers but not with intake by their fathers. This was consistent with the pattern in daughters aged 13–18 years (Tables S1–S3). Overall, the correlation for the dietary intake among siblings was higher than that for the intake of offspring with that of their parents. When our analysis was limited to the subgroup of subjects without diseases related to dietary control, the correlation between the overall diet of parents and offspring was slightly higher (*r* = 0.18, *p* < 0.0001), especially in mother–daughter pairs (*r* = 0.22, *p* < 0.0001) (data not shown), than the results presented in Table 2.

Associations between diet quality and variables of interest are presented in Table 3. Those with low-, middle-, and high-poor quality diets accounted for 48.5%, 43.5%, and 8.0% of the sample, respectively. Poor diet was associated with offspring’s age and sex, household socioeconomic status, and individual eating behaviors. The percentage of those with a high-poor diet increased as offspring matured from preschool to adolescence (5.1% among those 2–5 years, 33.1% at 6–12 years, and 61.9% at 13–18 years). With regard to the socioeconomic status of the household, consumption of a poor diet was significantly more common in households with the lowest income and in households in which both parents were less educated. Sharing any family meals, frequency of eating family meals per day, and eating breakfast were also significantly associated with diet quality, but eating out and parental smoking were not.

Multinomial logistic analysis was conducted to identify those factors that had independent effects on the diet of offspring (Table 4). Eating breakfast (*vs.* not doing so) showed a preventive effect against the risk for a poor diet, even after adjusting for relevant covariates (AOR 0.85, 95% CI 0.61–1.19 for middle-quality diet; AOR 0.54, 95% CI 0.33–0.90 for high-quality diet). Eating breakfast with family members and frequent family meals also showed preventive effects, but their impact was not significant after controlling for covariates.

**Table 1:** Basic characteristics of the sample.

Variables		Unit	Father (*n* = 1102)		Mother (*n* = 1239)		Son (*n* = 1020)		Daughter (*n* = 949)	
			Weighted Mean	S.E	Weighted Mean	S.E	Weighted Mean	S.E	Weighted Mean	S.E
Age		years	41.92	0.24	38.95	0.24	10.73	0.21	10.76	0.24
BMI		kg/m^2^	24.38	0.12	23.12	0.14	19.34	0.15	18.71	0.18
Average nutrient intake										
	Total calories	kcal	2568.51	32.46	1790.04	23.87	2078.03	35.55	1758.50	30.30
	Protein	g	95.86	1.57	66.39	1.17	75.78	1.82	61.86	1.25
	Vitamin A	μgRE	1057.04	38.83	803.71	29.05	705.32	44.13	566.17	27.68
	Vitamin B1	mg	1.75	0.03	1.24	0.03	1.43	0.03	1.20	0.03
	Vitamin B2	mg	1.61	0.03	1.19	0.03	1.42	0.03	1.19	0.02
	Vitamin C	mg	127.96	3.39	114.25	3.81	91.36	3.48	84.04	4.11
	Niacin	mg	22.74	0.39	15.74	0.30	15.81	0.39	13.13	0.30
	Calcium	mg	631.91	12.84	495.82	12.05	562.14	14.58	480.05	12.42
	Iron	mg	19.03	0.49	13.90	0.47	12.59	0.41	10.85	0.38
	Phosphorus	mg	1515.56	20.73	1108.45	17.87	1224.23	23.72	1017.41	18.51
Percentage with insufficient intake										
	Protein		2.49	0.50	4.92	0.79	0.82	0.44	2.95	0.71
	Vitamin A	%	46.05	1.82	46.28	1.92	54.17	2.00	55.86	2.08
	Vitamin B1	%	21.87	1.41	36.26	1.57	13.43	1.42	29.56	1.86
	Vitamin B2	%	56.75	1.68	49.20	1.94	39.78	2.04	34.35	1.93
	Vitamin C	%	48.98	1.71	51.54	1.70	57.22	2.18	61.38	2.12
	Niacin	%	21.86	1.46	33.35	1.55	27.64	1.85	36.72	2.09
	Calcium	%	77.56	1.36	74.47	1.60	81.76	1.47	86.72	1.25
	Iron	%	10.61	1.07	58.01	1.62	55.62	2.09	65.95	2.07
	Phosphorus	%	0.26	0.14	0.94	0.34	10.61	1.11	14.51	1.42
No. of insufficient nutrients		No.	2.86	0.06	3.55	0.08	3.41	0.08	3.88	0.08

S.E, standard error. The results for parametric variables are presented as weighted means with standard errors, and those for non-parametric variables are presented as weighted percentages with standard errors. These calculations considered the multi-stage sampling design of the KNHANES survey.

**Table 2:** Correlations of nutritional intake patterns among family members.

Nutrient	Familial Relationship									
	P-O	F-S	M-S	F-D	M-D	sib-sib	bro-bro	bro-sis	sis-sis	Spouse
Protein	0.00	−0.01	−0.02	0.01	0.01	0.23 ^d^	-	0.36 ^d^	−0.02	0.06 ^a^
Vitamin A	0.17 ^d^	0.16 ^d^	0.21 ^d^	0.14 ^d^	0.18 ^d^	0.36 ^d^	0.33 ^d^	0.40 ^d^	0.29 ^d^	0.22 ^d^
Vitamin B1	0.13 ^d^	0.14 ^d^	0.15 ^d^	0.09 ^b^	0.18 ^d^	0.24 ^d^	0.25 ^c^	0.21 ^d^	0.33 ^d^	0.16 ^d^
Vitamin B2	0.12 ^d^	0.07 ^a^	0.18 ^d^	0.08 ^a^	0.14 ^d^	0.29 ^d^	0.38 ^d^	0.25 ^d^	0.22 ^b^	0.18 ^d^
Vitamin C	0.20 ^d^	0.16 ^d^	0.23 ^d^	0.13 ^d^	0.27 ^d^	0.46 ^d^	0.44 ^d^	0.45 ^d^	0.48 ^d^	0.19 ^d^
Niacin	0.16 ^d^	0.14 ^d^	0.17 ^d^	0.09 ^b^	0.22 ^d^	0.36 ^d^	0.32 ^d^	0.34 ^d^	0.41 ^d^	0.14 ^d^
Calcium	0.09 ^d^	0.09 ^b^	0.14 ^d^	0.07 ^a^	0.06	0.33 ^d^	0.30 ^d^	0.34 ^d^	0.37 ^d^	0.09 ^b^
Iron	0.13 ^d^	0.08 ^b^	0.22 ^d^	0.03	0.19 ^d^	0.33 ^d^	0.34 ^d^	0.35 ^d^	0.28 ^d^	0.10 ^c^
Phosphorus	0.03 ^a^	0.03	0.08 ^b^	−0.02	0.04	0.14 ^d^	0.11	0.24 ^d^	0.00	−0.01
No. of insufficient nutrients	0.17 ^d^	0.16 ^d^	0.24 ^d^	0.09 ^a^	0.19 ^d^	0.43 ^d^	0.48 ^d^	0.43 ^d^	0.35 ^d^	0.18 ^d^

P-O, parents and offspring; F-S, father and son; M-S, mother and son; F-D, father and daughter; M-D, mother and daughter; sib-sib, sibling and sibling; bro-bro, brother and brother; bro-sis, brother and sister; sis-sis, sister and sister. a < 0.05, b < 0.01, c < 0.001, d < 0.0001. Correlation coefficient obtained using the FCOR package in the Statistical Analysis for Genetic Epidemiology software package. “-”, Correlation coefficient was not estimated due to the low prevalence of those with an insufficient intake of specific nutrients.

**Table 3:** Associations of diet quality with demographic characteristics, socioeconomic status, parental smoking, and eating behaviors in children and adolescents.

Variables	Subcategory	Number of Insufficient Nutrients						*p*-Value
		0–3 (Low)		4–6 (Mid)		7–9 (High)		
		Weighted %	S.E	Weighted %	S.E	Weighted %	S.E	
Age	2–5 years	30.16	1.98	8.58	1.09	5.10	2.15	<0.0001
	6–12 years	33.22	1.85	34.47	2.30	33.05	4.47	
	13–18 years	36.62	2.41	56.95	2.42	61.85	4.78	
Sex	boys	57.96	1.76	49.74	2.04	34.17	4.97	<0.0001
	girls	42.04	1.76	50.26	2.04	65.83	4.97	
Household income	Q1 (low)	4.50	0.97	8.66	1.89	7.77	3.57	0.02
	Q2	30.71	2.55	34.94	3.03	19.66	4.34	
	Q3	36.50	2.27	29.98	2.57	36.27	5.52	
	Q4 (high)	28.30	2.11	26.42	2.62	36.31	5.72	
Paternal education	≤12 years of schooling	39.45	2.50	51.18	3.15	42.93	6.19	<0.01
	13 years or more of schooling	60.55	2.50	48.82	3.15	57.07	6.19	
Maternal education	≤12 years of schooling	45.49	2.56	62.76	2.71	62.65	5.40	<0.0001
	13 years or more of schooling	54.51	2.56	37.24	2.71	37.35	5.40	
Paternal smoking	Yes	45.91	2.40	48.22	2.77	57.49	5.98	0.16
	No	54.09	2.40	51.78	2.77	42.51	5.98	
Maternal smoking	Yes	3.30	0.83	4.11	1.09	2.93	1.64	0.71
	No	96.70	0.83	95.89	1.09	97.07	1.64	
Eating meals with family members	Yes	89.02	1.40	84.39	1.91	80.01	4.13	0.03
	No	10.98	1.40	15.61	1.91	19.99	4.13	
Eating breakfast with family members	Yes	70.51	2.21	62.47	2.55	54.41	5.21	<0.01
	No	29.49	2.21	37.53	2.55	45.60	5.21	
Eating dinner with family members	Yes	80.48	2.01	71.91	2.45	65.96	4.99	<0.01
	No	19.52	2.01	28.09	2.45	34.04	4.99	
Frequency of eating meals with family members per day	0/day	10.98	1.40	15.61	1.91	19.99	4.13	<0.0001
	1/day	26.21	2.14	34.14	2.42	37.67	4.97	
	2/day	58.15	2.42	48.29	2.61	40.71	4.92	
	3/day	4.66	0.68	1.96	0.53	1.63	0.81	
Eating breakfast	Yes	82.38	1.65	74.06	1.96	66.50	4.98	<0.001
	No	17.62	1.65	25.94	1.96	33.50	4.98	
Eating out	≥once/day	25.34	2.21	25.97	2.17	33.49	5.02	0.30
	<none	74.66	2.21	74.03	2.17	66.52	5.02	

INQ: Index of Nutritional Quality; S.E; standard error. Poor diet quality was categorized according to the number of nutrients with insufficient intake as low (0–3), middle (4–6), and high (7–9).

**Table 4:** Multinomial logistic regression analysis of the risk for a poor diet according to eating behavior in children and adolescents.

Eating Behavior	Subcategory	No. of Insufficient Nutrients					
		4–6 (Mid)			7–9 (High)		
		AOR ^a^	95% CI		AOR ^a^	95% CI	
Eating meals with family members	Yes (ref. no)	1.07	0.72	1.60	0.76	0.38	1.52
Eating breakfast with family members	Yes (ref. no)	0.87	0.66	1.15	0.62	0.37	1.04
Eating dinner with family members	Yes (ref. no)	1.06	0.74	1.52	0.80	0.44	1.47
Frequency of eating meals with family members per day	0/day	0.80	0.36	1.80	1.11	0.32	3.81
	1/day	0.94	0.43	2.03	1.02	0.33	3.20
	2/day (ref. 3/day)	0.78	0.38	1.61	0.67	0.22	2.03
Eating breakfast	Yes (ref. no)	0.85	0.61	1.19	0.54	0.33	0.90
Eating out ^a^	≥once/day (ref. none)	0.79	0.56	1.12	1.19	0.71	1.97

INQ: Index of Nutritional Quality; AOR: adjusted odds ratio; 95% CI: 95% confidence interval. Poor diet quality was categorized according to the number of nutrients with insufficient intake as low (0–3), middle (4–6), and high (7–9). ^a^ Adjusted odds ratios were calculated after controlling for sex, age (2–5 year/6–12 year/13–18 year), maternal educational level (≤12 years of schooling/ 13 years or more of schooling), and quartile of household income. Reference group: the number of nutrients with insufficient intake: 0–3 (low).

## 4. Discussion

Due to changes in our food environment, the consumption of adequate levels of nutrients has become especially important for the maintenance of a healthy lifestyle that promotes longevity. Family environment makes an important contribution to the ability of children to develop healthy eating behaviors. Using Korean national data, this study showed that insufficient micronutrient intake was common and that such insufficiency was shared among family members. Furthermore, eating breakfast was significantly associated with better nutrition among children and adolescents.

It is known that scorbutic characteristics, night blindness, and iron-deficiency anemia are caused by deficiencies in dietary micronutrients such as vitamins and irons. Additionally, insufficient nutrient intake can lead to various chronic diseases, including obesity [2,3]. A lack of micronutrients is responsible for some of the disease burden borne by developing countries, including malnutrition. Furthermore, inadequate micronutrient intake is a problem even in developed countries that have abundant nutritional resources [8]. Micronutrients support various metabolic functions as well as the immune system and the cognitive development of children [3]. Some studies have reported insufficient micronutrient intake in children [7,8,12,15], adults [8,12], and older persons [8,12], but few have investigated associations among family members in this regard. Using a design similar to that employed in the current study, the US Department of Agriculture reported similarities between diet quality scores on the Healthy Eating Index (HEI) obtained by parents and those obtained by their children [9], but that study did not address micronutrients. The associations in the micronutrient intake patterns among family members found by the current study are consistent with those reported for macronutrients by a recent systematic review based on 15 studies (*r* = 0.2 for fat, 95% CI 0.13–0.28; *r* = 0.21 for total energy, 95% CI 0.18–0.24) [6]. Weaker associations than expected between data from parents and from offspring suggest that factors beyond the household environment play a role in this relationship. Indeed, we found stronger associations in the nutrient intake patterns among siblings than in those between parents and offspring, which may reflect age-related food preferences.

Overall, we found that insufficient intake of vitamin A, vitamin C, and calcium was common. Fruit and vegetables (F&V), milk, and dairy products are the main food sources for these nutrients. An increase in the proportion of individuals who do not meet the recommended criteria for F&V has been recently reported in the US [16], and a substantial portion of the Korean population did not meet the recommended criterion for F&V [17]. This may be responsible for the high prevalence of insufficient intake of micronutrients. Additionally, several studies have reported associations of F&V intake patterns among family members [6], which may partly account for the correlations with regard to micronutrient intake. However, this differed among studies. Despite the fact that calcium plays a key role in bone metabolism, including in the density of the bone minerals of growing children and in the prevention of osteoporosis in older people, we found substantial proportions of both offspring and parents with insufficient intake. The prevalence of insufficient intake was increased in children as well as parents, which is consistent with previous studies [8,13]. Although two cups (for children) and one cup (for adults) of milk per day were recommended by the KDRI, this average intake was not reached by most respondents in that study [13]. Thus, it is urgent that we develop strategies to ameliorate these situations.

We also examined the dietary status of children and adolescents according to the socioeconomic status of their households, their parents’ smoking status, and their own eating behaviors. Indeed, it is possible that socioeconomic status, including parental education and household income, may influence food choices due to differences in nutrient-related knowledge, the priority accorded to health considerations, and the cost of food [5,8,17,18]. A study using the 2005 KNHANES data found that individual INQ values significantly differed according to paternal educational level, but this was not considered in the design of the KNHANES sampling method [12]. Another study, conducted in Nova Scotia, also reported that higher parental educational level was associated with a lower risk of a poor diet in children aged 10–11 years [15]. Consistent with the above studies, we found a negative association between socioeconomic status and poor diet, but this association disappeared in the multinomial logistic analysis. This result may be attributable to the close association between household socioeconomic status and the eating behaviors of children (e.g., family meals, eating breakfast, and eating out). A study of low-income African-Americans found that unhealthy maternal behaviors (e.g., smoking) and obesity influenced the frequency of family meals and snacking by children [5]. However, we found no association between parental smoking and the quality of the diet of offspring.

Although interventions made when children are young are effective, their influence on children of different ages and sexes varies according to household environment. The fact that younger children tend to remain with a guardian, primarily their mother, for a long period of the day may account for the significant associations between offspring dietary behaviors and those of mothers, but not fathers. In contrast, the association between the diet quality of offspring and parents was weakest in adolescents, and it was significant only in adolescent females. The reduction in the frequency of family meals and the increase in eating choices during adolescence may have contributed to these weak correlations [9]. One study found that adolescent boys ate out more frequently than adolescent girls did [19]. We found no sex differences in the frequency of eating out (≥once per day) (13–18 years: boys, 40.2% *vs.* girls, 38.6%; *p* = 0.71), but the finding regarding sex differences in participation in family meals was only borderline significant (13–18 years: boys, 69.6% *vs.* girls, 77.0%; *p* = 0.06). Furthermore, accumulated evidence indicates that the eating behaviors of adolescents tend to be affected by peers and that this effect differs by sex [5]. Thus, appropriate strategies should be based on considerations of the sex and age of the target group.

Several studies have found that the frequency of family meals was associated with the quality of the diet consumed. Veugelers *et al.* [15] reported that frequent family suppers was independently associated with a lower risk for a poor diet, whereas skipping breakfast was associated with an approximately 18% increase in the risk for a poor diet in children. The attenuated effect of family meals after controlling for socioeconomic status may be associated with the aforementioned issues. The relationship between socioeconomic status and diet quality, through eating behaviors, requires additional study. Nevertheless, the present study showed a significant effect of eating breakfast, and this remained even after adjusting for the body mass index of the child (AOR 0.54, 95% CI: 0.33–0.90). Indeed, extant evidence suggests that eating breakfast is positively associated with scores for dietary variety [20] and dietary quality [7]. In contrast, less frequent consumption of breakfast has been associated with snacking [7]. The proportion of those who skipped breakfast increased with age in both sexes (boys: 10.09% at 2–5 years, 11.17% at 6–12 years, and 35.99% at 13–18 years, *p* for trend <0.0001; girls: 11.62% in those aged 2–5 years, 11.57% in those aged 6–12 years, and 34.12% in those aged 13–18 years, *p* for trend <0.0001). These data may help explain the high proportion of adolescents with poor diets. Consistent with a previous study [7], we found that those who ate breakfast were less likely than those who did not to consume an insufficient quantity of each nutrient (data not shown). Eating breakfast may reflect the overall quality of the diet consumed by children and adolescents. Several recent epidemiological studies have indicated that the frequency of eating breakfast regularly has been gradually decreasing over time and that it follows a negative trend as a function of age [21]. Taken together with these previous results, our data underscore the importance of public health campaigns that encourage people to eat breakfast.

This study has several limitations. First, the use of a single measure may have increased variation among individuals and reduced reliability. Although some studies have reported insufficient micronutrient status using dietary indicators found in blood and urine, the problem of measurement error remains. However, 24-h dietary recall data are reliable for assessing current nutritional status, and the use of trained interviewers to collect data reduced the possibility of error due to information bias. As assessments of diet quality vary according to the tool used, opportunities for direct comparisons with other studies are limited. The INQ is an easy way to assess nutritional quality in terms of nutritional density, and it can reveal imbalances between nutrient and caloric intake. However, as we assessed only a limited number of micronutrients, further studies are required. Nevertheless, our study has several strengths. First, to maximize the accuracy of our calculations, we excluded respondents with dietary limitations due to medical conditions. However, the effect of this variable was not significant. Second, our use of nationally representative data allows for generalization. Third, the KNHANES is conducted throughout the year, rendering it unaffected by seasonal variations. Fourth, we attempted to avoid the attenuated effect of familial environment by restricting our sample to offspring aged 2–18 years. Finally, our approach that examined the quality rather than simply the quantity of nutrients consumed may provide an opportunity to identify practical approaches to dietary problems.

Our results provide valuable information that was previously lacking and support the need to develop strategies to improve the quality of diets consumed by Koreans. The primary targets for such efforts should consist of mothers with preschool children and adolescents, who would benefit from peer- or school-based interventions. Although the establishment of healthy eating behaviors in childhood is important for preventing diseases [22], the lack of effective approaches to improving the quality of the diets consumed by children and to ameliorating their unhealthy eating behaviors persists. Thus, additional studies are required.

## Supplementary Materials

Table S1: Correlations of nutritional intake patterns among family members in households with children aged 2–5 years.

Table S2: Correlations of insufficient nutrition intakes among family members in household with children aged 6–12 years.

Table S3: Correlations of insufficient nutrition intakes among family members in household with children aged 13–18 years.

Figure S1: Weighted percentages of Korean children with insufficient nutrient intake by age and sex.

## References

[1] Kim S., Moon S., Popkin B.M. (2000). The nutrition transition in South Korea. Am. J. Clin. Nutr..

[2] Lee H.A., Kim Y.J., Lee H., Gwak H.S., Park E.A., Cho S.J., Oh S.Y., Ha E.H., Kim H.S., Park H. (2013). Association of vitamin D concentrations with adiposity indices among preadolescent children in Korea. J. Pediatr. Endocrinol. Metab..

[3] Shenkin A. (2006). Micronutrients in health and disease. Postgrad. Med. J..

[4] Xu J., Eilat-Adar S., Loria C., Goldbourt U., Howard B.V., Fabsitz R.R., Zephier E.M., Mattil C., Lee E.T. (2006). Dietary fat intake and risk of coronary heart disease: The strong heart study. Am. J. Clin. Nutr..

[5] Wang Y., Li J., Caballero B. (2009). Resemblance in dietary intakes between urban low-income African-American adolescents and their mothers: The healthy eating and active lifestyles from school to home for kids study. J. Am. Diet. Assoc..

[6] Wang Y., Beydoun M.A., Li J., Liu Y., Moreno L.A. (2011). Do children and their parents eat a similar diet? Resemblance in child and parental dietary intake: Systematic review and meta-analysis. J. Epidemiol. Community Health.

[7] Park H.A., Kang J.H., Kim K.W., Cho Y.G., Hur Y.I., Kim O.H. (2011). Breakfast skipping, related factors, and nutrients intake of 5th grade students. Korean J. Fam. Med..

[8] Manios Y., Moschonis G., Mavrogianni C., Bos R., Singh-Povel C. (2014). Micronutrient intakes among children and adults in Greece: The role of age, sex and socio-economic status. Nutrients.

[9] Beydoun M.A., Wang Y. (2009). Parent-child dietary intake resemblance in the United States: Evidence from a large representative survey. Soc. Sci. Med..

[10] Kweon S., Kim Y., Jang M.J., Kim Y., Kim K., Choi S., Chun C., Khang Y.H., Oh K. (2014). Data resource profile: The Korea National Health and Nutrition Examination Survey (KNHANES). Int. J. Epidemiol..

[11] The Korean Nutrition Society (2010). Dietary reference intakes for Koreans.

[12] Kim K., Hong S.A., Kim M.K. (2008). Nutritional status and food insufficiency of Korean population through the life-course by education level based on 2005 national health and nutrition survey. Korean J. Nutr..

[13] Shim J.S., Oh J., Nam C.M. (2008). Association of household food security with dietary intake—Based on the Third (2005) Korea National Health and Nutrition Examination Survey (KNHANES III). Korean J. Nutr..

[14] The S.A.G.E Project S.A.G.E.—Statistical Analysis for Genetic Epidemiology. http://darwin.cwru.edu/sage/.

[15] Veugelers P.J., Fitzgerald A.L., Johnston E. (2005). Dietary intake and risk factors for poor diet quality among children in Nova Scotia. Can. J. Public Health.

[16] Centers for Disease Control and Prevention MMWR September 2010 State–Specific Trends in Fruit and Vegetable Consumption among Adults United States, 2000–2009. http://www.cdc.gov/mmwr/pdf/wk/mm5935.pdf.

[17] Hong S.A., Kim K., Kim M.K. (2012). Trends in the inequality of fruit and vegetable consumption between education levels indicated by the Korea National Health and Nutrition Examination Surveys. Eur. J. Clin. Nutr..

[18] Ramachandran A., Snehalatha C. (2010). Rising burden of obesity in Asia. J. Obes..

[19] Burke V., Beilin L.J., Dunbar D. (2001). Family lifestyle and parental body mass index as predictors of body mass index in Australian children: A longitudinal study. Int. J. Obes. Relat. Metab. Disord. J. Int. Assoc. Study Obes..

[20] Kang M.H., Lee J.S., Kim H.Y., Kwon S., Choi Y.S., Chung H.R., Kwak T.K., Cho Y.H. (2012). Selection items of a food behavior checklist for the development of nutrition quotient (NQ) for children. Korean J. Nutr..

[21] Alexy U., Wicher M., Kersting M. (2010). Breakfast trends in children and adolescents: Frequency and quality. Public Health Nutr..

[22] Faith M.S., Keller K.L., Johnson S.L., Pietrobelli A., Matz P.E., Must S., Jorge M.A., Cooperberg J., Heymsfield S.B., Allison D.B. (2004). Familial aggregation of energy intake in children. Am. J. Clin. Nutr..

