# Supplementary material for: Correlations between Poor Micronutrition in Family Members and Potential Risk Factors for Poor Diet in Children and Adolescents Using Korean National Health and Nutrition Examination Survey Data
Source: Nutrients. 2015 Aug 4;7(8):6346–61. doi: 10.3390/nu7085286 (PMC4555125; doi:10.3390/nu7085286)
Supplement: Supplementary file 1 [file nutrients-07-05286-s001.doc]

Supplementary Information

**Table S1.** Correlations of nutritional intake patterns among family members in households with children aged 2–5 years.

| **Nutrients** | **Familial Relationship** | | | | | | | | | |
| --- | --- | --- | --- | --- | --- | --- | --- | --- | --- | --- |
|  | **P-O** | **F-S** | **M-S** | **F-D** | **M-D** | **sib-sib** | **bro-bro** | **bro-sis** | **sis-sis** | **Spouse** |
| Protein | - | - | - | - | - | - | - | - | - | −0.03 |
| Vitamin A | 0.22 ^d^ | 0.12 | 0.32 ^d^ | 0.18 ^b^ | 0.27 ^d^ | 0.37 ^c^ | 0.16 | 0.51 ^c^ | 0.38 | 0.21 ^d^ |
| Vitamin B1 | 0.09 ^b^ | −0.01 | 0.07 | 0.12 | 0.16 ^a^ | 0.15 | −0.10 | −0.09 | 0.61 ^c^ | 0.11 ^a^ |
| Vitamin B2 | 0.15 ^d^ | 0.10 | 0.25 ^d^ | 0.10 | 0.17 ^b^ | 0.45 ^d^ | - | 0.51 ^c^ | 0.32 | 0.21 ^d^ |
| Vitamin C | 0.20 ^d^ | 0.15 ^a^ | 0.29 ^d^ | 0.15 ^a^ | 0.22 ^c^ | 0.52 ^d^ | 0.46 | 0.48 ^c^ | 0.61 ^c^ | 0.23 ^d^ |
| Niacin | 0.11 ^b^ | −0.04 | 0.11 | 0.11 | 0.23 ^c^ | 0.29 ^b^ | −0.17 | 0.40 ^b^ | 0.29 | 0.09 |
| Calcium | 0.09 ^b^ | 0.11 | 0.19 ^b^ | −0.01 | 0.08 | 0.50 ^d^ | 0.02 | 0.55 ^d^ | 0.67 ^d^ | 0.09 |
| Iron | 0.12 ^c^ | 0.10 | 0.20 ^b^ | 0.05 | 0.21 ^c^ | 0.40 ^c^ | −0.04 | 0.49 ^c^ | 0.43 ^a^ | 0.11 ^a^ |
| Phosphorus | 0.05 ^a^ | −0.13 | 0.20 ^d^ | −0.02 | −0.02 | −0.04 | - | - | −0.14 | −0.01 |
| No. of insufficient nutrients | 0.14 ^d^ | 0.03 | 0.22 ^c^ | 0.11 | 0.22 ^c^ | 0.33 ^b^ | 0.03 | 0.38 ^b^ | 0.39 | 0.14 ^b^ |

P-O, parents and offspring; F-S, father and son; M-S, mother and son; F-D, father and daughter; M-D, mother and daughter; sib–sib, sibling and sibling; bro–bro, brother and brother; bro–sis, brother and sister; sis–sis, sister and sister. ^a^ < 0.05, ^b^ < 0.01, ^c^ < 0.001, ^d^ < 0.0001. Correlation coefficient obtained using the FCOR package in the Statistical Analysis for Genetic Epidemiology software package. “-”, Correlation coefficient was not estimated due to the low prevalence of those with an insufficient intake of specific nutrients.

**Table S2.** Correlations of insufficient nutrition intakes among family members in household with children aged 6–12 years.

| **Nutrients** | **Familial Correlation** | | | | | | | | | |
| --- | --- | --- | --- | --- | --- | --- | --- | --- | --- | --- |
|  | **P-O** | **F-S** | **M-S** | **F-D** | **M-D** | **sib-sib** | **bro-bro** | **bro-sis** | **sis-sis** | **Spouse** |
| Protein | −0.02 | −0.01 | −0.02 | −0.02 | 0.02 | −0.004 | - | −0.01 | - | 0.10 ^b^ |
| Vitamin A | 0.22 ^d^ | 0.22 ^d^ | 0.26 ^d^ | 0.21 ^d^ | 0.20 ^d^ | 0.28 ^d^ | 0.23 | 0.29 ^c^ | 0.34 ^a^ | 0.24 ^d^ |
| Vitamin B1 | 0.17 ^d^ | 0.19 ^d^ | 0.20 ^d^ | 0.07 | 0.23 ^d^ | 0.35 ^d^ | 0.17 | 0.42 ^d^ | 0.37 ^b^ | 0.23 ^d^ |
| Vitamin B2 | 0.12 ^d^ | −0.01 | 0.24 ^d^ | 0.11 ^a^ | 0.13 ^b^ | 0.30 ^d^ | 0.42 ^c^ | 0.21 ^a^ | 0.26 | 0.15 ^d^ |
| Vitamin C | 0.24 ^d^ | 0.16 ^c^ | 0.28 ^d^ | 0.19 ^c^ | 0.34 ^d^ | 0.52 ^d^ | 0.44 ^c^ | 0.50 ^d^ | 0.70 ^d^ | 0.18 ^d^ |

**Table S2.** *Cont.*

| **Nutrients** | **Familial Correlation** | | | | | | | | | |
| --- | --- | --- | --- | --- | --- | --- | --- | --- | --- | --- |
|  | **P-O** | **F-S** | **M-S** | **F-D** | **M-D** | **sib-sib** | **bro-bro** | **bro-sis** | **sis-sis** | **Spouse** |
| Niacin | 0.19 ^d^ | 0.20 ^d^ | 0.20 ^d^ | 0.08 | 0.27 ^d^ | 0.45 ^d^ | 0.44 ^d^ | 0.41 ^d^ | 0.63 ^d^ | 0.17 ^d^ |
| Calcium | 0.09 ^c^ | 0.09 ^a^ | 0.13 ^b^ | 0.15 ^b^ | −0.003 | 0.27 ^d^ | 0.32 ^b^ | 0.24 ^b^ | 0.30 ^a^ | 0.08 ^a^ |
| Iron | 0.16 ^d^ | 0.09 | 0.25 ^d^ | 0.06 | 0.26 ^d^ | 0.35 ^d^ | 0.39 ^b^ | 0.35 ^d^ | 0.29 ^a^ | 0.08 ^a^ |
| Phosphorus | 0.02 | 0.10 ^b^ | 0.05 | −0.04 | - | 0.19 ^d^ | 0.33 ^c^ | 0.21 ^c^ | −0.13 | −0.01 |
| No. of insufficient nutrients | 0.23 ^d^ | 0.19 ^d^ | 0.31 ^d^ | 0.17 ^c^ | 0.25 ^d^ | 0.41 ^d^ | 0.50 ^d^ | 0.40 ^d^ | 0.36 ^b^ | 0.19 ^d^ |

P-O, parents and offspring; F-S, father and son; M-S, mother and son; F-D, father and daughter; M-D, mother and daughter; sib-sib, sibling and sibling; bro-bro, brother and brother; bro-sis, brother and sister; sis-sis, sister and sister. ^a^ < 0.05, ^b^ < 0.01, ^c^ < 0.001, ^d^ < 0.0001. Correlation coefficient obtained using the FCOR package in the Statistical Analysis for Genetic Epidemiology software package. “-”, Correlation coefficient was not estimated due to the low prevalence of those with an insufficient intake of specific nutrients.

**Table S3.** Correlations of insufficient nutrition intakes among family members in household with children aged 13–18 years.

| **Nutrients** | **Familial Correlation** | | | | | | | | | |
| --- | --- | --- | --- | --- | --- | --- | --- | --- | --- | --- |
|  | **P-O** | **F-S** | **M-S** | **F-D** | **M-D** | **sib-sib** | **bro-bro** | **bro-sis** | **sis-sis** | **Spouse** |
| Protein | 0.01 | −0.02 | −0.03 | 0.01 | 0.04 | 0.32 ^c^ | - | 0.70 ^d^ | −0.05 | 0.11 ^a^ |
| Vitamin A | 0.08 ^a^ | 0.10 | 0.07 | 0.04 | 0.12 ^a^ | 0.24 ^b^ | 0.39 ^a^ | 0.23 | 0.22 | 0.16 ^c^ |
| Vitamin B1 | 0.12 ^c^ | 0.15 ^b^ | 0.16 ^b^ | 0.11 | 0.16 ^b^ | 0.23 ^b^ | 0.20 | 0.19 | 0.29 | 0.18 ^d^ |
| Vitamin B2 | 0.10 ^b^ | 0.09 | 0.08 | 0.06 | 0.18 ^b^ | 0.16 | 0.24 | 0.22 | −0.03 | 0.13 ^b^ |
| Vitamin C | 0.17 ^d^ | 0.19 ^c^ | 0.13 ^a^ | 0.09 | 0.25 ^d^ | 0.36 ^d^ | 0.49 ^b^ | 0.27 ^a^ | 0.30 | 0.20 ^d^ |
| Niacin | 0.15 ^d^ | 0.18 ^b^ | 0.19 ^b^ | 0.05 | 0.14 ^a^ | 0.20 ^a^ | 0.17 | 0.23 | 0.18 | 0.17 ^c^ |
| Calcium | 0.11^c^ | 0.06 | 0.12 ^a^ | 0.13 ^a^ | 0.15 ^b^ | 0.16 | 0.39 ^a^ | 0.10 | 0.15 | 0.08 |

**Table S3. *Cont.***

| **Nutrients** | **Familial Correlation** | | | | | | | | | |
| --- | --- | --- | --- | --- | --- | --- | --- | --- | --- | --- |
|  | **P-O** | **F-S** | **M-S** | **F-D** | **M-D** | **sib-sib** | **bro-bro** | **bro-sis** | **sis-sis** | **Spouse** |
| Iron | 0.12 ^d^ | 0.01 | 0.25 ^d^ | 0.04 | 0.15 ^a^ | 0.26 ^b^ | 0.13 | 0.43 ^c^ | 0.25 | 0.04 |
| Phosphorus | 0.04 | −0.02 | 0.08 | - | 0.14 ^a^ | 0.02 | −0.07 | 0.02 | 0.03 | −0.01 |
| No. of insufficient nutrients | 0.11 ^c^ | 0.19 ^b^ | 0.13 ^a^ | 0.06 | 0.13 ^a^ | 0.26 ^b^ | 0.34 | 0.28 ^a^ | 0.16 | 0.18 ^d^ |

P-O, parents and offspring; F-S, father and son; M-S, mother and son; F-D, father and daughter; M-D, mother and daughter; sib-sib, sibling and sibling; bro-bro, brother and brother; bro-sis, brother and sister; sis-sis, sister and sister. ^a^ < 0.05, ^b^ < 0.01, ^c^ < 0.001, ^d^ < 0.0001. Correlation coefficient obtained using the FCOR package in the Statistical Analysis for Genetic Epidemiology software package. “-”, Correlation coefficient was not estimated due to the low prevalence of those with an insufficient intake of specific nutrients.

**Figure S1.** Weighted percentages of Korean children with insufficient nutrient intake by age and sex. ^a^ *P* < 0.01 for age differences; ^b^ *P* < 0.0001 for age differences. Intake of each nutrient per 1000 kcal of total energy by age and sex and recommended intake of each nutrient per 1000 kcal. The weighted percentages of those with insufficient intake of each nutrient were calculated in consideration of the sampling method used by the Korean National Health and Nutrition Examination Survey. The weighted percentages of Korean children with insufficient nutrient intake were calculated by age group (squares: 2–5 years; circles: 6–12 years old; diamonds: 13–18 years), where (A) indicates boys, and (B) indicates girls.
